# Meta-analysis of plate fixation versus intramedullary fixation for the treatment of mid-shaft clavicle fractures

**DOI:** 10.1186/s13049-015-0108-0

**Published:** 2015-03-20

**Authors:** Bing Zhang, Yanbin Zhu, Fei Zhang, Wei Chen, Ye Tian, Yingze Zhang

**Affiliations:** Department of Orthopaedic Surgery, The Third Hospital of Hebei Medical University, NO.139 Ziqiang Road, Shijiazhuang, Hebei 050051 P.R. China; Department of Hand Surgery, The Third Hospital of Hebei Medical University, Shijiazhuang, Hebei 050051 P.R. China; Key Laboratory of Biomechanics of Hebei Province, Shijiazhuang, Hebei 050051 P.R. China

**Keywords:** Mid-shaft clavicle fractures, Intramedullary fixation, Plate fixation, Meta-analysis

## Abstract

**Background:**

This systematic review and meta-analysis aims to critically compare the outcomes of plate fixation (PF) versus intramedullary fixation (IF) for the treatment of mid-shaft clavicle fractures.

**Methods:**

Relevant original studies were searched in electronic databases of Medline, Embase, Cochrane central database and CNKI (all through October 2014). The study was performed according to the PRISMA statement. Studies that investigated the comparing effectiveness or complications between both groups and provided sufficient data of interest were included in this meta-analysis.

**Results:**

Thirteen studies fulfilling inclusion and exclusion criteria were included in this meta-analysis, which included 479 participants in PF group and 457 in IF group**.** Compared to PFs, IFs outperformed PFs associated with a reduced surgery time, a shorter incision, rapid union time, better shoulder function recovery at 6 months and fewer complications of symptomatic hardware, refracture after hardware removal and hypertrophic scar. In other aspects such as functional recovery at 12 months and 24 months follow-up, shoulder motion range, complications of superficial infection,temporary brachial plexus lesion, nonunion, malunion, delayed union, implant failure, major revision needed, both techniques were comparable.

**Conclusions:**

The present evidence from this meta-analysis suggested that IF was a more advantaged method for the treatment of midshaft clavicle fractures. This present study might aid surgeons in making evidence-based decision about optimal surgical treatment of mid-shaft clavicular fracture.

**Electronic supplementary material:**

The online version of this article (doi:10.1186/s13049-015-0108-0) contains supplementary material, which is available to authorized users.

## Introduction

Clavicular fracture accounts for 2.6–10% of all fractures, and approximately 80% of the sites involved in adult patients were mid-shaft clavicle [1-4]. Furthermore, displacement occurs in over 70% of the mid-shaft clavicular fractures [4,5]. Traditionally, non-operative treatment has been labeled as the “standard” for mid-shaft fractures regardless of displacement, with the expectation that even severe radiographic malalignment would not influence functional results [6]. However recently, increasing evidence has challenged this due to the relatively high incidence of complications, deficits in functional recovery in shoulder and disappointing cosmetic results in up to 30% of the patients sustaining mid-shaft clavicular fracture [7-9]. With recent advancement in technique and implants for fracture fixation, internal fixation is therefore generally considered as the better choice for these fractures and admirable outcomes have been observed. However, substantial controversies exist in surgeons regarding the optimal fixation pattern (plate or intramedullary fixation) for treating these injuries and further research is necessitated.

To date, there are various techniques for fixation of mid-shaft clavicle fractures including multiple forms of plating and intramedullary devices. For plating fixation (PF), precontoured locking plates (DCP) [10,11] and reconstruction plate [12-14] are the most commonly used devices. For intramedullary fixation (IF), the common devices in clinics are Knowles pinning [14,15], elastic stable intramedullary nailing [12,16], Rockwood Clavicle Pin (RCP) [17,18] and Acumed Clavicle Rod (ACR) [17]. PF emerged as a popular technique affording a more rigid fixation that is necessitated for early rehabilitation protocols [19]. Recently, precontoured plates that are designed to parallel the S-shaped curve of the clavicle have become popular alternatives. However, the advantages are compromised by extensive soft tissue dissection that potentially result in damage to the superior clavicular nerves and subsequent paresthesias, implant prominence, infection, scarring, hardware failure and refracture after the removal of the plate [20-23]. On the other hand, IF advantaged over PF in preserving the soft tissue envelope, periosteum, and vascular integrity [24,25] and even early hardware migration appears to be solved by improved device of locked IM, but the biomechanical property of less stability has to be addressed [26].

Houwert et al [27] and Barlow et [28] conducted systematic reviews on related articles comparing both methods with equivocal conclusions. Duan et al [29] conducted a meta-analysis with RCT studies, but only 2 studies were included to investigate the comparative outcomes between both methods. From then on, several original studies have been performed to address this key issue [10,14,17,21,30,31], which necessitated this updated meta-analysis.

Therefore, the purpose of this study is to evaluate whether one method of fixation is preferable over the other in terms of intraoperative variables, postoperative complications, shoulder motion and functional recovery for the treatment of acute mid-shaft clavicle fractures.

## Materials and methods

### Search strategy

A computerized literature search was initially performed in databases of PubMed, Medline, Embase and Chinese National Knowledge Infrastructure (CNKI) for related studies published between January 1990 and October 2014. The main keywords were as follows: “clavicular” or “clavicle” AND “mid-shaft” AND “fracture” AND “intramedullary” AND “plate” or “plating” AND “complications” or “effectiveness” or “results” or “outcome”. A manual search of references was also performed in the original articles and systematic reviews for additional inclusion.

### Inclusion criteria

Using the criteria of the PRISMA statement, two reviewers (Zhang F and Tian Y) independently evaluated the titles and abstracts of the identified papers [32]. Only full-text articles without language restriction were included in this meta-analysis. Disagreements were discussed, and if not resolved a third reviewer was consulted. The inclusion criteria were as follows: (1) randomised controlled trials or case–control studies or cohort studies comparing the results between IF and PF for treating mid-shaft clavicular fractures; (2) at least one of comparing results of interest must be provided in the original study; (3) age of participants in both groups was not older than 65 years, namely, young active patients; (4) sufficient data were provided for estimating an odds ratio (OR) or standardized mean difference (SMD) with 95% confidence intervals (CIs).

Studies were excluded if they evaluated patients with multitrauma, or patients undergoing surgery for a revision, infection, or non- or-malunion.

### Quality assessment and data extraction

As the included studies were various in methodology, including observational or experimental and prospective or retrospective; therefore, no precise scale could be performed to assess the quality of them. This problem was solved by a tool recommended in the Cochrane Handbook 5.1.0, as “The Cochrane Collaboration’s tool for assessing risk of bias”, which was used by Fang et al [33] to solve the similar problem. This scale includes 6 major concerns as bias sources including sequence generation, allocation concealment, blinding, incomplete data, selective outcome reporting and others. All the data were carefully and independently extracted from all eligible studies by the same two reviewers (Zhang F and Tian Y). The following basic characteristics were extracted from each study: first author’s name, publication year, patients’ age and gender, follow-up duration, definitions and numbers of IF and PF groups, numbers of citations for each observed item.

### Statistical analysis

ORs and corresponding 95% CI were estimated and pooled across studies to assess the differences between the both methods with a *P* < 0.05 as significance. Heterogeneity among studies was tested by Q-test statistics with significance set at *P* < 0.10 [34] and further measured by *I*^2^ statistics with *I*^2^ more than 50% indicating significant inconsistency. A random-effect model was used to calculate pooled ORs in the case of significant heterogeneity (*P* < 0.10 or *I*^2^ > 50%); otherwise, a fixed-effect model was used [35]. The outcome of meta-analysis for variables was summarized graphically using a forest plot. Publication bias was assessed and graphed by funnel plot. For any variable presenting with large heterogeneity, a sensitive analysis by excluding outlier studies was conducted to investigate the sources for heterogeneity. All analyses were performed using the software Stata 11.0 (Stata Corporation, College Station, TX).

## Results

### Research results and basic information

**Figure 1:**
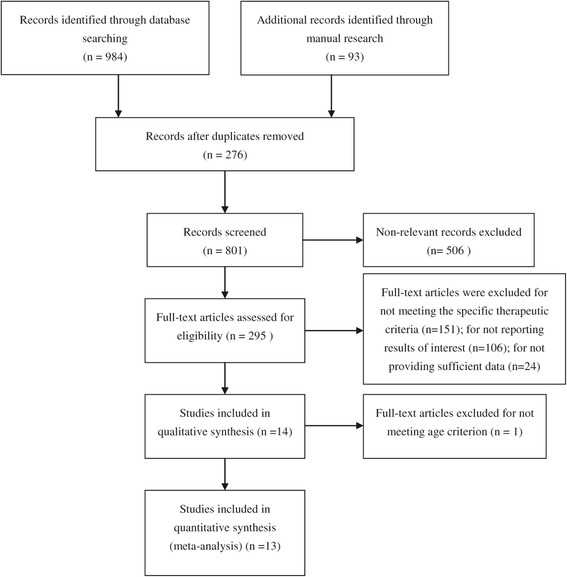
**Flow diagram of literature searching.**

The initial database search yielded 1077 papers, 783 were excluded due to inappropriate types (e.g. abstracts, reduplicative, letters and meeting reports); 151 were excluded for not meeting the specific therapeutic methods according to criteria; 106 were excluded for reporting results of no interest; 24 were excluded as they did not provide sufficient data for meta-analysis; 1 study was excluded owing to not fulfilling the age criteria [15]; and finally, 13 studies were identified to be eligible and included in this meta-analysis. The whole research procedure was presented by a flow diagram (Figure 1).

In the 13 comparative original articles included, 12 were published in English with publication time from 2008 to 2014 and only 1 in Korean published in 1999. These studies included 457 participants in the IF group and 479 in the PF group, respectively. As described in each study, patients treated by both methods were comparable in term of gender, side invloved and injury mechanism. However, regarding admission age, two studies reported significantly higher age in PF than IF group [31,36] and in other studies the age between both groups were comparable. Participants in twelve studies were followed up at least a mean of 12 months and in one study the follow-up time was a mean of 5.9 months [37]. A summary of basic characteristics was listed in Table 1. The detailed information of quality assessment for included studies was presented in Additional file 1: Table S1.

**Table 1:** **Detailed information on the basic characteristics of the 13 included studies and participants**

**Author**	**Country (area)**	**Publication year**	**RCT or Not**	**IF (case)**	**PF (case)**	**IF age (mean)**	**PF age (mean)**	**IF (M/F)**	**PF (M/F)**	**Follow-up (months)**
Narsaria [10]	India	2014	RCT	33	32	38.9 ± 9.1 (20–62)	40.2 ± 11.2 (18–64)	24/9	26/6	>24
Liu [12]	China,Taiwan	2010	Not	51	59	33.6 ± 13.5	31.7 ± 9.7	32/19	29/30	>12
Fu [14]	China,Taiwan	2012	Not	53	50	35.2 ± 14.5	39.9 ± 14.8	38/15	33/17	>12
Ko [38]	Korea	1999	Not	13	18	36.6 (23–68)	43.3 (18–74	NP	NP	>12
Lee J [17]	USA	2014	Not	43	67	27.6 (14–59)	31.7 (16–68)	29/5	63/4	>15
Wenninger [30]	USA	2013	Not	33	29	25.2 (18–51)	26.9 (20–49)	32/1	26/3	>12
Assobhi [36]	Egypt	2011	RCT	19	19	30.3 ± 4.8 (24–45)	32.6 ± 5.9 (26–49)	16/3	17/2	>12
Wijdicks [31]	The Netherlands	2012	Not	47	43	33.1 ± 15.6	39.4 ± 14.1	33/14	33/10	>12
Ferran [40]	UK	2010	RCT	17	15	23.8 (13–42)	35.4 (16–53)	14/3	13/2	12.4
(Mean)										
Kleweno [41]	USA	2011	Not	18	14	35 (16–56)	28 (16–46)	15/3	10/4	>12
Chen [21]	China	2012	Not	57	84	34.3 (20–59)	36.5 (19–63)	41/16	61/23	>24
Lee Y [24]	China,Taiwan	2008	RCT	56	32	40.1	38.2	37/19	20/12	>12
Thyagarajan [37]	UK	2009	Not	17	17	28 (15–56)	32.1 (17–46)	16/1	15/2	5.9

*Abbreviation: RCT* randomized controlled trials, *PF* plate fixation, *IF* intramedullary fixation, *M* males, *F* females.

### Intraoperative and perioperative variables

**Figure 2:**
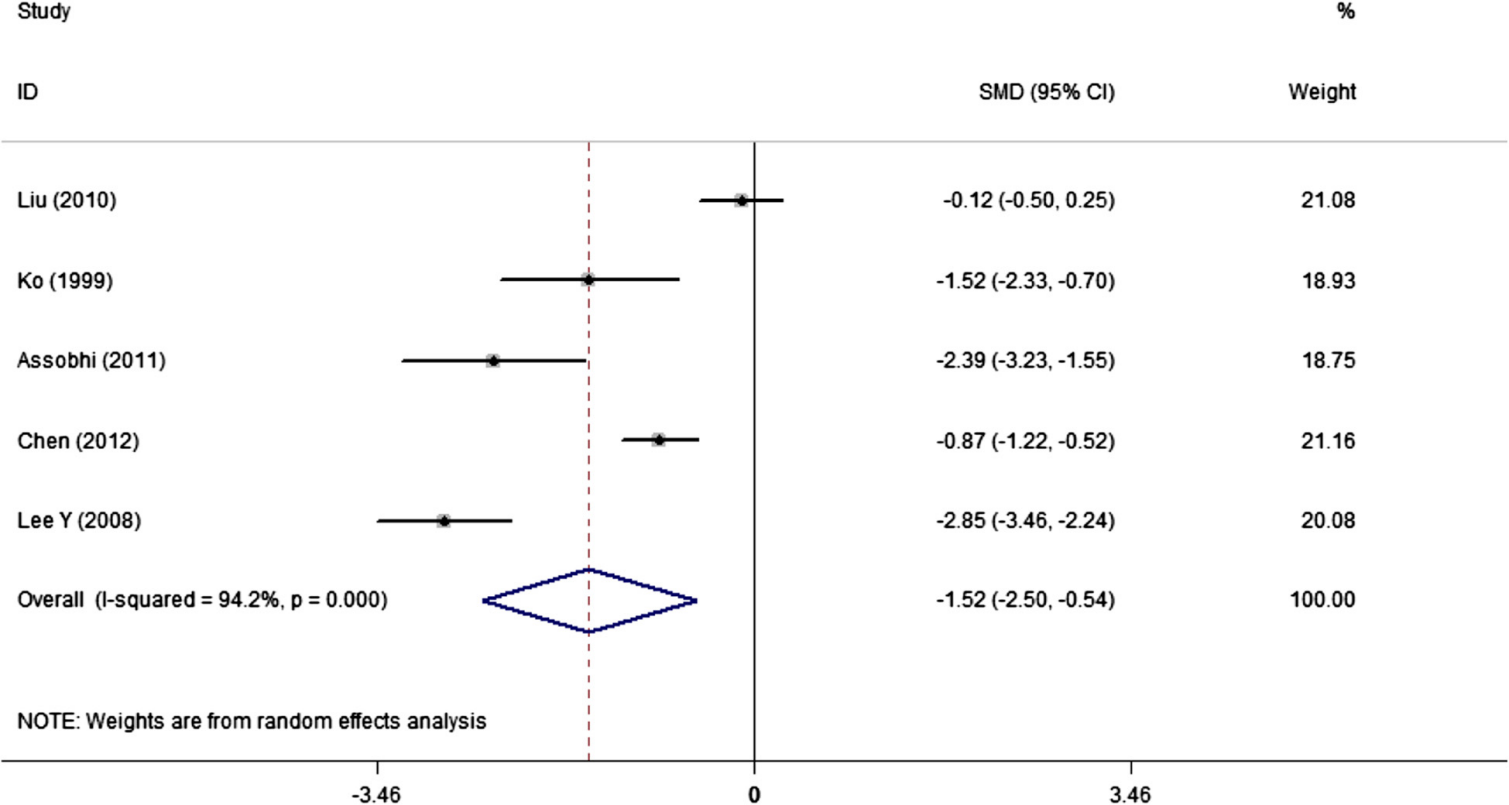
**Forest plot of SMD with 95%CI for surgery time (in favor of IF).**

**Figure 3:**
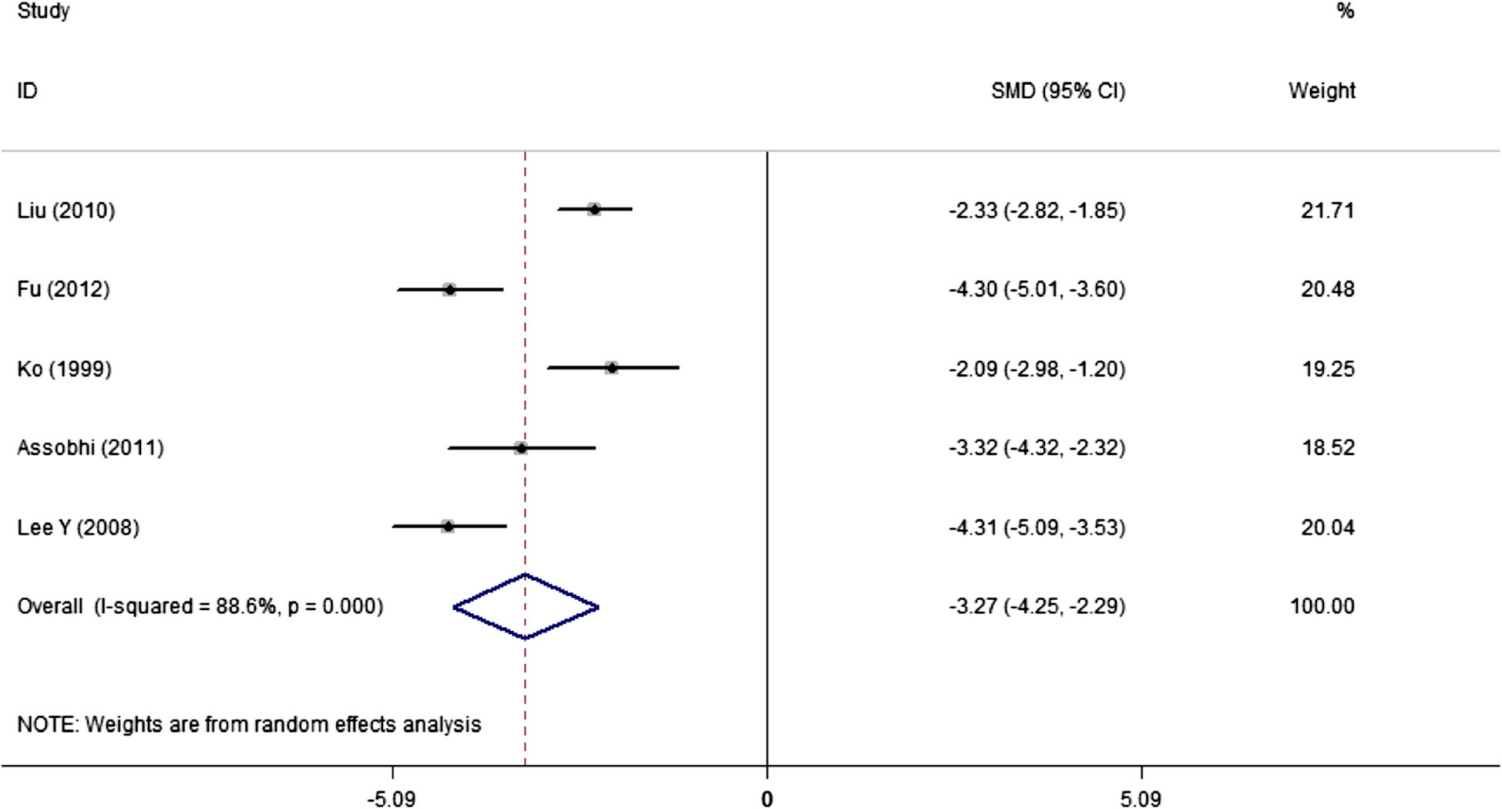
**Forest plot of SMD with 95%CI for the analysis of incision length (in favor of IF).**

**Figure 4:**
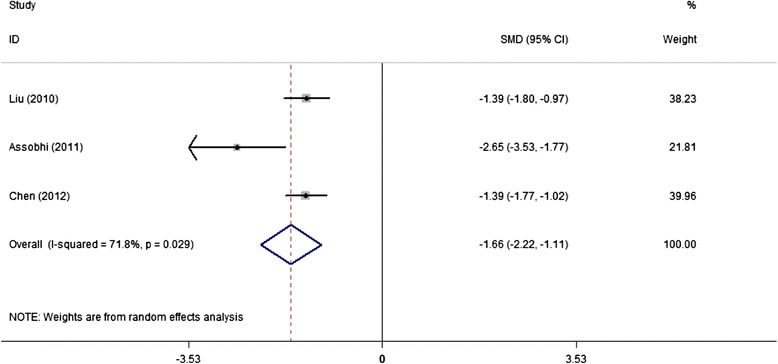
**Forest plot of SMD with 95%CI for the analysis of blood loss (in favor of IF).**

Seven studies reported surgery time, with means of 53.0 and 77.8 minutes in IF and PF groups, respectively. Five studies could provide standard data form of mean and standard deviation (sd), the discrepancy approached to significance (SMD,-1.52; 95% CI,-2.50 to-0.54) but with large heterogeneity (*I*^2^ = 94.2%) (Figure 2). However, we did not perform sensitive analysis due to the reported significant difference for surgery time in each study, and therefore this pooled result was believed to be reliable. Similarly, IF advantaged over PF groups with smaller incision (5.8 cm VS 11.4 cm) and less blood loss (67.0 ml VS 130.0 ml), reported by each of the 5 and 4 studies, respectively. The combined results favored the original reported result but with significant heterogeneity as I^2^ was 88.6% for incision length and 71.8% for blood loss (Figures 3 and 4).

### Postoperative complications

**Figure 5:**
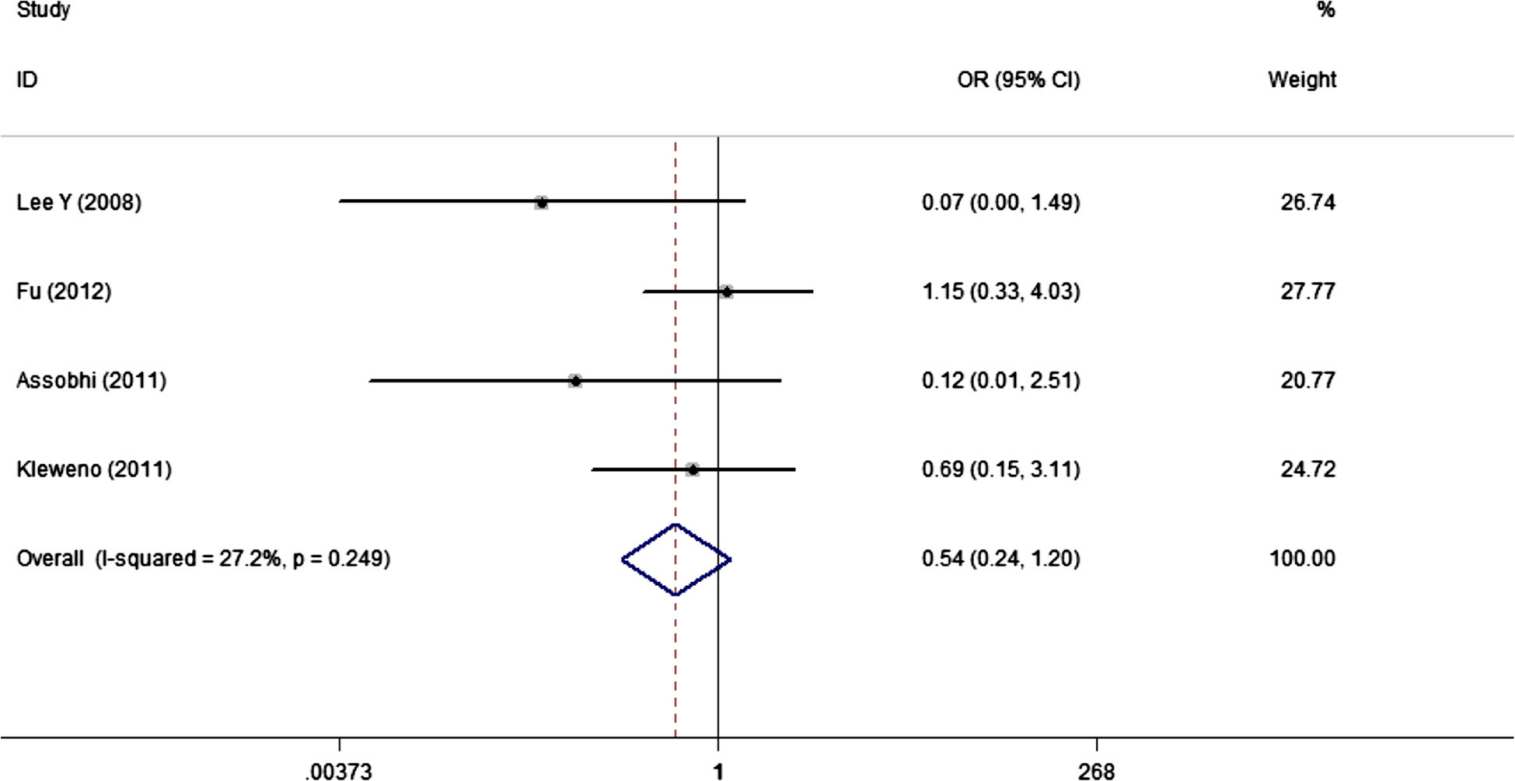
**Forest plot of OR with 95%CI for the incidence of total complications.**

Four studies reported the overall complications, although with a slightly higher incidence rate in PF than IF groups (13.9% VS 7.5%) the combined result did not reach significance (OR, 0.54; 95%CI, 0.24 to 1.20) (Figure 5).

**Figure 6:**
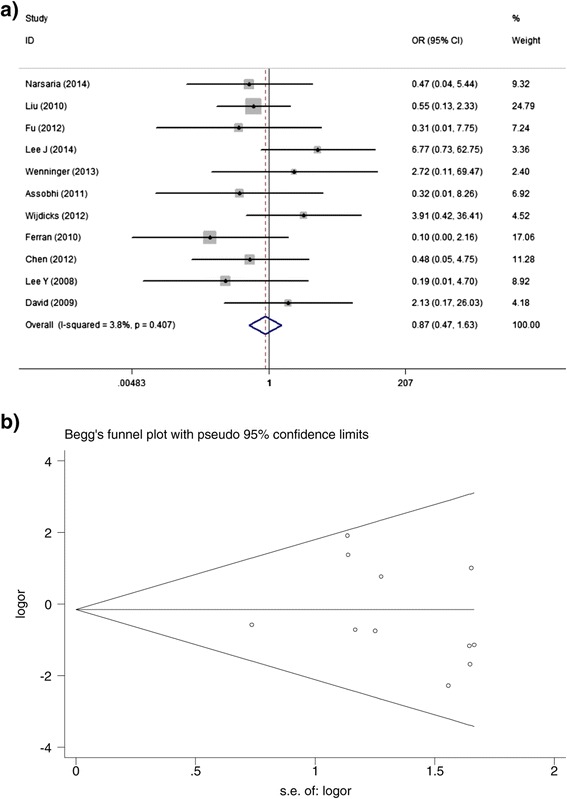
**Plots of meta-analysis results for the incidence of superficial infection. a**. Forest plot of OR with corrosponding 95%CI. **b**. Funnel plot for the analysis of publication bias using Begg’s test.

Eleven studies involving 426 IFs and 447 PFs paid close attention to postoperative superficial infection, the meta-analysis did not investigate a significant difference between both methods (OR, 0.87; 95%CI, 0.47 to 1.63) (Figure 6a) with little heterogeneity (I^2^ = 3.8%). Begg’s test showed no publication bias for this variable and the result was graphed by funnel plot (Figure 6b).

**Figure 7:**
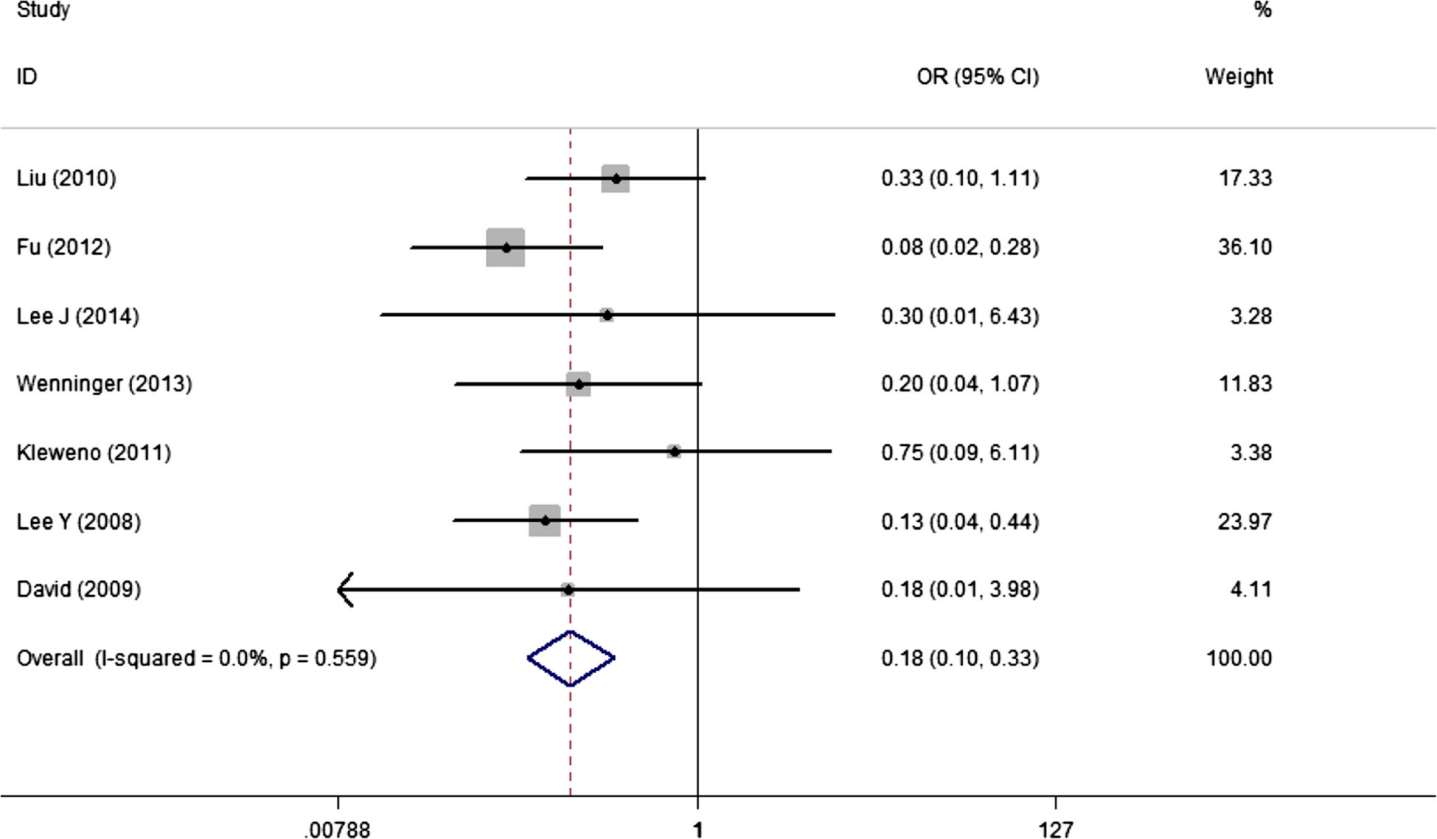
**Forest plot of OR with 95%CI for the incidence of postoperative symptomatic hardware (in favor of IF).**

Seven studies involving 268 IFs and 271 PFs reported the incidence of postoperative symptomatic hardware with a higher rate in PF group (22.0%) and lower in IF group (5.5%). The meta-analysis did investigate a significant difference without any heterogeneity (OR, 0.18; 95%CI, 0.10 to 0.33) (Figure 7).

**Figure 8:**
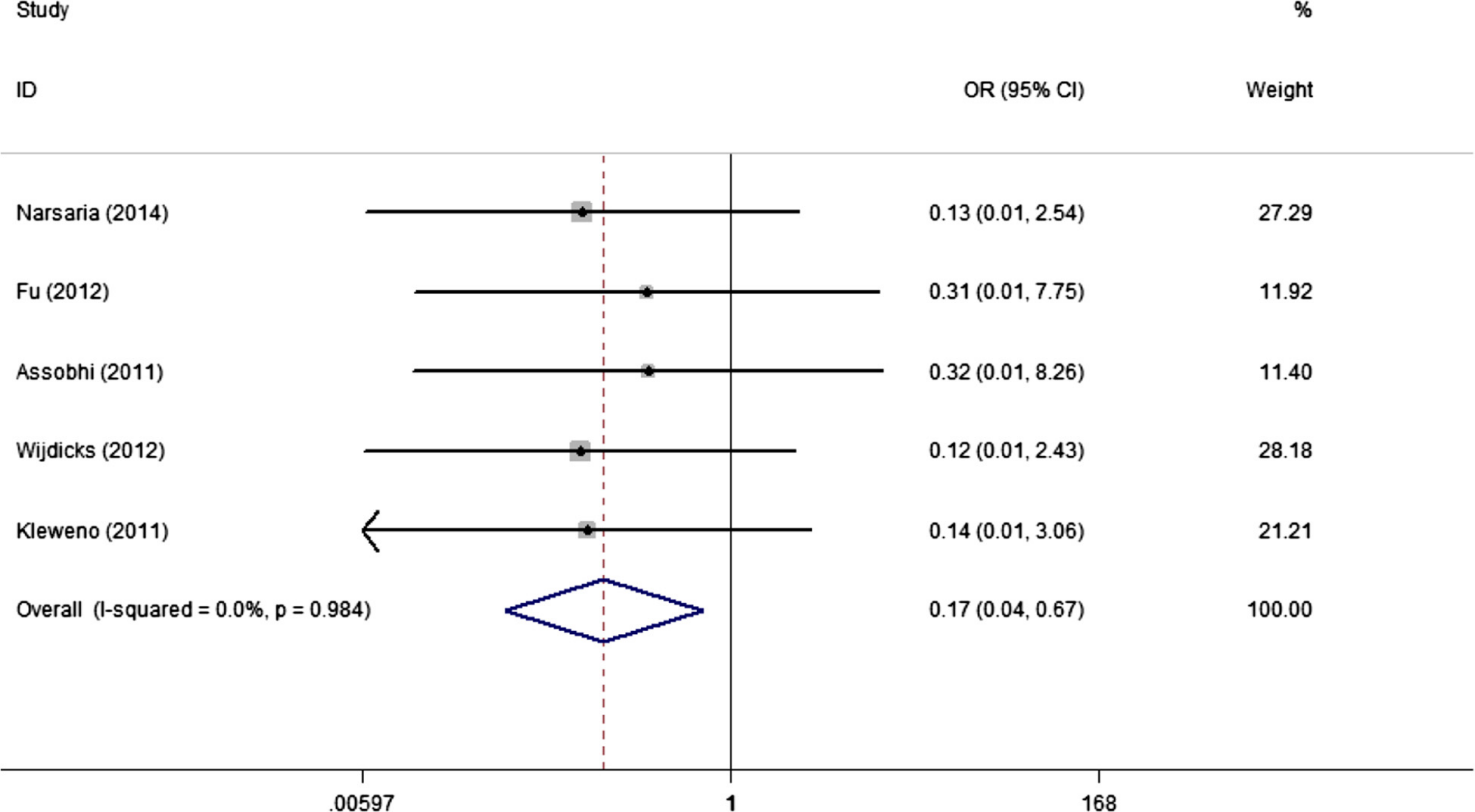
**Forest plot of OR with 95%CI for the incidence of refracture after implant removal (in favor of IF).**

**Figure 9:**
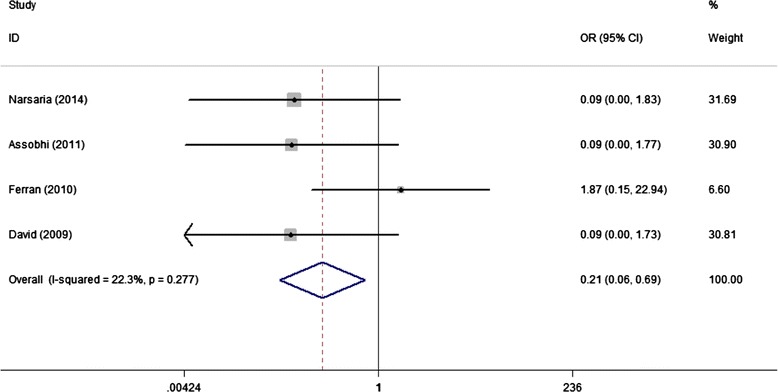
**Forest plot of OR with 95%CI for the incidence of hypertrophic scar (in favor of IF).**

Regarding other complications including refracture after implant removal and hypertrophic scar, IF advantaged over PF with lower incidences of 0% (VS 6.3%) and 2.3% (VS 15.7%), respectively; and the combined result approached to significance (Figures 8 and 9).

However, with regard to temporary brachial plexus lesion, nonunion, malunion, delayed union, implant failure, major revision needed, there were no significant differences observed between the two fixation methods. For any variable, no statistical significance in heterogeneity was investigated both using Q and I^2^ tests. The results were presented in Table 2.

**Table 2:** **Detailed data on 13 observational comparing variables between both methods and the outcomes of meta-analyses**

**Variables**	**No of studies**	**Pooled OR or SMD**	**LL 95% CI**	**UL 95% CI**	***P*** **-value**	**Q-test for heterogeneity (** ***P*** **)**	^**c**^ **I** ^**2**^ **(%)**
Implant failure	6	1.03^*^	0.43	2.48	0.94^a^	0.320	14.8
Nonunion	7	0.90^*^	0.44	1.84	0.78^a^	0.697	0
Malunion	4	1.32^*^	0.54	3.22	0.55^a^	0.478	0
Delayed union	2	0.20^*^	0.02	1.94	0.17^a^	0.890	0
Major revision needed	2	0.25^*^	0.05	1.24	0.09^a^	0.532	0
Temporary brachial plexus lesion	2	1.69^*^	0.31	9.19	0.54^a^	0.343	0
Shoulder motion							
Forward flexion	2	-0.10^#^	-0.43	0.23	0.55^a^	0.430	0
Abduction	2	-0.33^#^	-0.67	-0.004	0.05^a^	0.423	0
External rotation	2	-0.22^#^	-0.56	0.11	0.19^a^	0.855	0
Internal rotation	2	-0.32^#^	-0.66	0.01	0.06^a^	0.606	0
Constant scores							
6-mon	3	1.02^#^&	0.64	1.41	<0.001^a^&	0.230	32.6
12-mon	3	0.22^#^	-0.39	0.83	0.47^b^	0.033	70.8
24-mon	2	-0.21^#^	-0.82	0.39	0.50^b^	0.040	75.6

*Abbreviation: SMD* standardized mean difference, *OR* odds ratio, *LL* lower limit, *UL* upper limit.

^*^Pooled OR was used; ^#^Pooled SMD was used.

& Favor IF as advantage.

^a^Fixed-effects model was performed.

^b^Fandom-effects model was performed.

^c^I^2^ statistic was defined as the proportion of heterogeneity not due to chance or random error.

### Functional and motion range assessment

Two studies by Ko et al [38] and Liu et al [12] reported the shoulder motion range including forward flexion, abduction, external rotation and internal rotation. However, no significant difference was investigated and no heterogeneity was observed for any item; the results were presented in Table 2.

The Constant Shoulder Scores (CSS) were used to assess the extent of functional recovery with higher scores representing better rehabilitation. Based on original studies, CSS at 6-mon, 12-mon and 24-mon follow-up were reported by 3, 3 and 2 studies, respectively. At the 6-mon follow-up, patients could obtain a slightly higher CSS (94.3 VS 89.4) in IF group and the meta-analysis investigated the significance (SMD, 1.02; 95%CI, 0.64 to 1.41). However, at the 12-mon and 24-mon follow-up the significance disappeared, representing a comparable result between both groups (Table 2).

### Descriptive analysis

IFs were reported to be associated with a significantly shorter union time, but we are unable to perform the meta-analysis due to the significant heterogeneity. American Shoulder and Elbow Surgeons (ASES) score and Disabilities of the Arm, Shoulder,and Hand (DASH) were used to assess the shoulder function recovery at different follow-up stage. However, different data forms were provided or the scoring system at different follow-up stage was reported in a single study. Therefore, no meta-analysis was performed. For example, DASH score at 6-mon and 24-mon follow-up was reported by only 1 study [21] and PFs obtain higher scores than IFs. DASH score at 12-month follow-up was reported in 2 studies [12,38] and a slightly higher score was observed in IFs, but no meta-analysis was attempted due to the significant heterogeneity.

## Discussion

Management of acute mid-shaft clavicular fractures in patients has been undergoing controversy on which fixation pattern was preferable when making a clinic decision. We therefore performed this systematic and meta-analysis from 13 studies including 457 IFs and 479 PFs to the compare the effectiveness and complications between the both techniques. In this meta-analysis, IF advantaged over PF groups with reduced surgery time, smaller incision, less blood loss and better functional recovery at 6-mon follow-up postoperatively. Meanwhile, the shoulder motion range was not significantly different in term of forward flexion, abduction, external rotation and internal rotation. Regarding postoperative complications, IF was confirmed to be associated with lower incidence of superficial infection, symptomatic hardware, hypertrophic scar and refracture after implant removal while not increasing the risk of implant failure, nonunion, malunion, delayed union, major revision needed and temporary brachial plexus lesion.

In regard to perioperative variables including surgery time, incision length and blood loss, significant heterogeneity was investigated due to the various types of intramedullary devices and plates. However, we did not perform corresponding sensitive analysis because of the reported significance by each study for any item; therefore the pooled results could be reliable and convincing. Although IF did outperform PF in the functional recovery with a higher should constant score at 6-mon follow-up, no significance was observed at 12- and 24-mon follow-up. Furthermore, regarding shoulder motion range at the last follow-up in each study, IFs performed similarly as PFs. Therefore, the rapid fracture union might contribute primarily to the functional recovery and shoulder motion at early-stage but did not work at late-stage.

Patients’ complication status was commonly documented in the literature, which was determined by patients’ systemic conditions (underlying disease), the local operation and the implant fixation per se. In this meta-analysis, participants included in each original study were almost young (mean age from 23.8 to 43.3), and the study which investigated only patients aged above 65 years was excluded from this meta-analysis at the literature-search stage. Therefore, underlying diseases might contribute little to the incidence of complications and the major causes for complications were operation and fixation pattern.

It is notable that, the refracture after hardware removal occurred only in PFs with the incidence rate of 6.3% (10/158) but none in IFs. In the study by Wijdicks et al [31], two explanations are used by authors to clarify the causes: subsequent reduced mechanical strength after implant removal when fracture healing and the weak spots of the screw holes after implant removal both potentially initiated a refracture in thin clavicles. Implant failure is an important complication that necessitates secondary operation and causes over 80% of revisions [31], but no significant difference was observed between both fixations. Despite this, the common implant failure modes are different. Wijdicks et al [31] suggested intramedullary device’ movement restore the fracture segment to the original form and in PFs, the plate might bend or break due to excessive movement. Therefore, the favorable solutions for this might improve stability in IFs and reduce excessive movement in PFs especially in early-stage, postoperatively. In Harnroongroj’ and Lee’s studies, implant failure was attributed to the length of intramedullary pin engagement and small pin could provide better stability [17,39]. Authors suggested patients treated by PFs should gradually increase should motion range and keep within 90°during the first 3 weeks after surgery [21]. However, different plates and pins were used in above-mentioned studies. Therefore, how to effectively prevent this complication in certain fixations is worth surgeons’ consideration and patients’ cooperation.

The present study suffers from some weaknesses. Firstly, most of the studies (9/13) in this meta-analysis were retrospective, case-control studies and only 4 RCTs were included, which might lower the assessing quality of this study. Secondly, the types of fixations applied in studies were varied and the follow-up periods in studies ranged largely from several months to years. Thirdly, the age between both fixations in most studies was comparable but not in all the studies, which might affect the results. Although with limitations that might lead to heterogeneity, no significant heterogeneity was observed in most variables except for several intraoperative items, indicating the results reliable. Due to the relative lower quality of studies, the conclusion should be treated cautiously and further prospective studies with better design should be performed to verify the conclusions.

This study has own merits. First, the search style based on the computer and manual search ensures a complete inclusion of relevant studies. Secondly, this meta-analysis to date gives a definitive conclusion of preference for either of the 2 methods in treatment of mid-shaft fractures, reflecting the current status on this issue.

In conclusion, IF advantaged over PF groups with reduced surgery time, smaller incision, less blood loss and better functional recovery at 6-mon follow-up postoperatively. Meanwhile, fewer superficial infections, symptomatic hardware, hypertrophic scar and refracture after implant removal occurred in IFs. Clinical decision should be skewed to IFs if medical conditions are indicated.

## Additional file

Additional file 1: Table S1. Methodological assessment of the included articles for the analysis using the Cochrane Collaboration’s tool for assessing risk of bias.

## Abbreviations

PF: Plate fixation
IF: Intramedullary fixation
RCT: Randomized controlled trials
M: Males
F: Females
SMD: Standardized mean difference
OR: Odds ratio
LL: Lower limit
UL: Upper limit
