# Supplementary material for: Meta-analysis of plate fixation versus intramedullary fixation for the treatment of mid-shaft clavicle fractures
Source: Scand J Trauma Resusc Emerg Med. 2015 Mar 20;23:27. doi: 10.1186/s13049-015-0108-0 (PMC4372272; doi:10.1186/s13049-015-0108-0)
Supplement: Additional file 1: Table S1. — Methodological assessment of the included articles for the analysis using the Cochrane Collaboration’s tool for assessing risk of bias. [file 13049_2015_108_MOESM1_ESM.docx]

| References | Random sequence  generation | Allocation  concealment | Blinding | Incomplete  outcome data | Free of selective  reporting | Free of  other bias |
| --- | --- | --- | --- | --- | --- | --- |
| Narsaria 2014 | Yes | Yes | Unclear | Yes | Yes | Yes |
| Liu 2010 | No | Unclear | Yes | Yes | Yes | Unclear |
| Fu 2012 | No | Unclear | Yes | Yes | Yes | Unclear |
| Ko 1999 | Yes | Unclear | Unclear | Yes | Yes | No |
| Lee J 2014 | Unclear | Yes | Unclear | Yes | Yes | Yes |
| Wenninger 2013 | Yes | Unclear | Yes | Yes | Yes | Unclear |
| Assobhi 2011 | No | Yes | Yes | Unclear | Yes | No |
| Wijdicks 2012 | Yes | Yes | Yes | Yes | Yes | No |
| Ferran 2010 | No | Unclear | Unclear | Yes | Yes | Yes |
| Kleweno 2011 | Yes | Yes | Yes | Yes | Yes | No |
| Chen 2012 | No | Unclear | Yes | Yes | Yes | No |
| Lee Y 2008 | Yes | Yes | Unclear | Unclear | Yes | Yes |
| Thyagarajan 2009 | No | Yes | Yes | Yes | Yes | Yes |

**Additional file 1: Table S1** Methodological assessment of the included articles for the analysis using the Cochrane Collaboration’s tool for assessing risk of bias.

Explanation: ‘Yes’ indicates low risk of bias, ‘Unclear’ indicates unclear risk of bias, ‘No’ indicates high risk of bias
